# Apoptosis dysfunction: unravelling the interplay between ZBP1 activation and viral invasion in innate immune responses

**DOI:** 10.1186/s12964-024-01531-y

**Published:** 2024-02-24

**Authors:** Jianhao Zhan, Jisheng Wang, Yuqing Liang, Lisha Wang, Le Huang, Shanshan Liu, Xiaoping Zeng, Erming Zeng, Hongmei Wang

**Affiliations:** 1 Department of Neurosurgery, the First Affiliated Hospital of Nanchang University, Nanchang, Jiangxi Province 330006 China; 2 HuanKui Academy, Nanchang University, Nanchang, Jiangxi Province 330006 China; 3 School of Basic Medical Sciences, Nanchang University, Nanchang, Jiangxi Province 330006 China; 4 Medical College, Jinhua Polytechnic, Jinhua, Zhejiang Province 321017 China

**Keywords:** ZBP1, Viral invasion, Apoptosis inhibition, Innate immunity

## Abstract

Apoptosis plays a pivotal role in pathogen elimination and maintaining homeostasis. However, viruses have evolved strategies to evade apoptosis, enabling their persistence within the host. Z-DNA binding protein 1 (ZBP1) is a potent innate immune sensor that detects cytoplasmic nucleic acids and activates the innate immune response to clear pathogens. When apoptosis is inhibited by viral invasion, ZBP1 can be activated to compensate for the effect of apoptosis by triggering an innate immune response. This review examined the mechanisms of apoptosis inhibition and ZBP1 activation during viral invasion. The authors outlined the mechanisms of ZBP1-induced type I interferon, pyroptosis and necroptosis, as well as the crosstalk between ZBP1 and the cGAS-STING signalling pathway. Furthermore, ZBP1 can reverse the suppression of apoptotic signals induced by viruses. Intriguingly, a positive feedback loop exists in the ZBP1 signalling pathway, which intensifies the innate immune response while triggering a cytokine storm, leading to tissue and organ damage. The prudent use of ZBP1, which is a double-edged sword, has significant clinical implications for treating infections and inflammation.

## Introduction

Apoptosis, which is also known as programmed cell death, represents a fundamental defence mechanism used by the immune system to eliminate infected or damaged cells, including those targeted by pathogens [1, 2]. Throughout the infection process, apoptosis serves as a means to restrict pathogen replication, prevent the spread of infection, and maintain tissue homeostasis [3, 4]. During apoptosis, the infected or compromised cell undergoes a series of molecular events, leading to controlled cell death and subsequent removal by phagocytes [5]. However, in the ongoing evolutionary struggle between viruses and the host, viruses, which are obligate intracellular parasites, have evolved sophisticated strategies to counteract apoptosis and promote their own survival; these strategies include simulating the function of the antiapoptotic protein B-cell lymphoma-2 protein (Bcl-2) [6], terminating death receptor signalling, and inhibiting the activity of caspases [7]. These viral evasion mechanisms aim to prolong the lifespan of infected cells, facilitating viral replication and dissemination within the host.

Therefore, when apoptosis cannot play a role, how can cells clear invading pathogens? The innate immune response is as a robust mechanism through which the body can counteract pathogens. Host cells recognize pathogen-associated molecular patterns (PAMPs) through pathogen recognition receptors (PRRs) on the membrane surface or within the cytoplasm. This recognition triggers downstream signalling pathways, resulting in the expression of interferon (IFN), chemokines, and proinflammatory cytokines, ultimately initiating innate immune responses to eradicate pathogens [8]. When apoptosis is inhibited, host cells can clear pathogens by activating the innate immune response. Moreover, host cells can trigger alternative modes of cell death. These other forms of cell death can effectively contribute to the clearance of invading pathogens, thereby compensating for the role of apoptosis in pathogen clearance [9].

Innate immune sensors play important roles in this process. To counteract viral infections, the host immune system has evolved various innate immune sensors to detect viral invasion and initiate immune responses. One sensor is Z-DNA binding protein 1 (ZBP1), also known as DNA-dependent activator of IFN-regulatory factors (DAI) or DLM-1. It acts as a cytoplasmic innate immune sensor that can detect viral nucleic acids and trigger downstream signalling pathways to combat viral infections [10]. In recent years, an increasing number of studies have revealed that ZBP1 plays an important role in inhibiting viral infection and innate immunity.

In this review, we examine into the intricate mechanisms underlying apoptosis failure in the presence of invading pathogens. Additionally, we explored the pathways by which cells activate ZBP1 in response to pathogen invasion, leading to apoptosis failure. We discuss the process through which activated ZBP1 initiates innate immune responses to aid in the clearance of pathogens and the preservation of internal homeostasis.

## Mechanism by which viruses inhibit cell apoptosis

A conventional understanding of apoptosis regulation indicates two primary pathways: the extrinsic death receptor pathway and the intrinsic mitochondrial pathway. The death receptor pathway is initiated by the binding of ligands, such as Fas-L and TNF-α, to their corresponding receptors. In response to ligand‒receptor interaction, cells initiate apoptotic signals, leading to the activation of intracellular caspases, including caspase-3, caspase-6, and caspase-7 [11–13]. On the other hand, the mitochondrial pathway, which is known as the intrinsic pathway, is triggered by various stress factors, including DNA damage, oxidative stress, and endoplasmic reticulum stress [14]. Subsequently, Bcl-2-associated X protein (BAX) and Bcl2 antagonist/killer (BAK) are activated, forming pores in the mitochondrial outer membrane and inducing mitochondrial outer membrane permeabilization (MOMP). This results in the release of mitochondrial matrix contents into the cytoplasm [15], which can activate caspases by forming apoptotic complexes [16]. These effector caspases, which can be activated by either pathway, can cleave various downstream cellular components, ultimately inducing apoptosis [17].

**Fig. 1:**
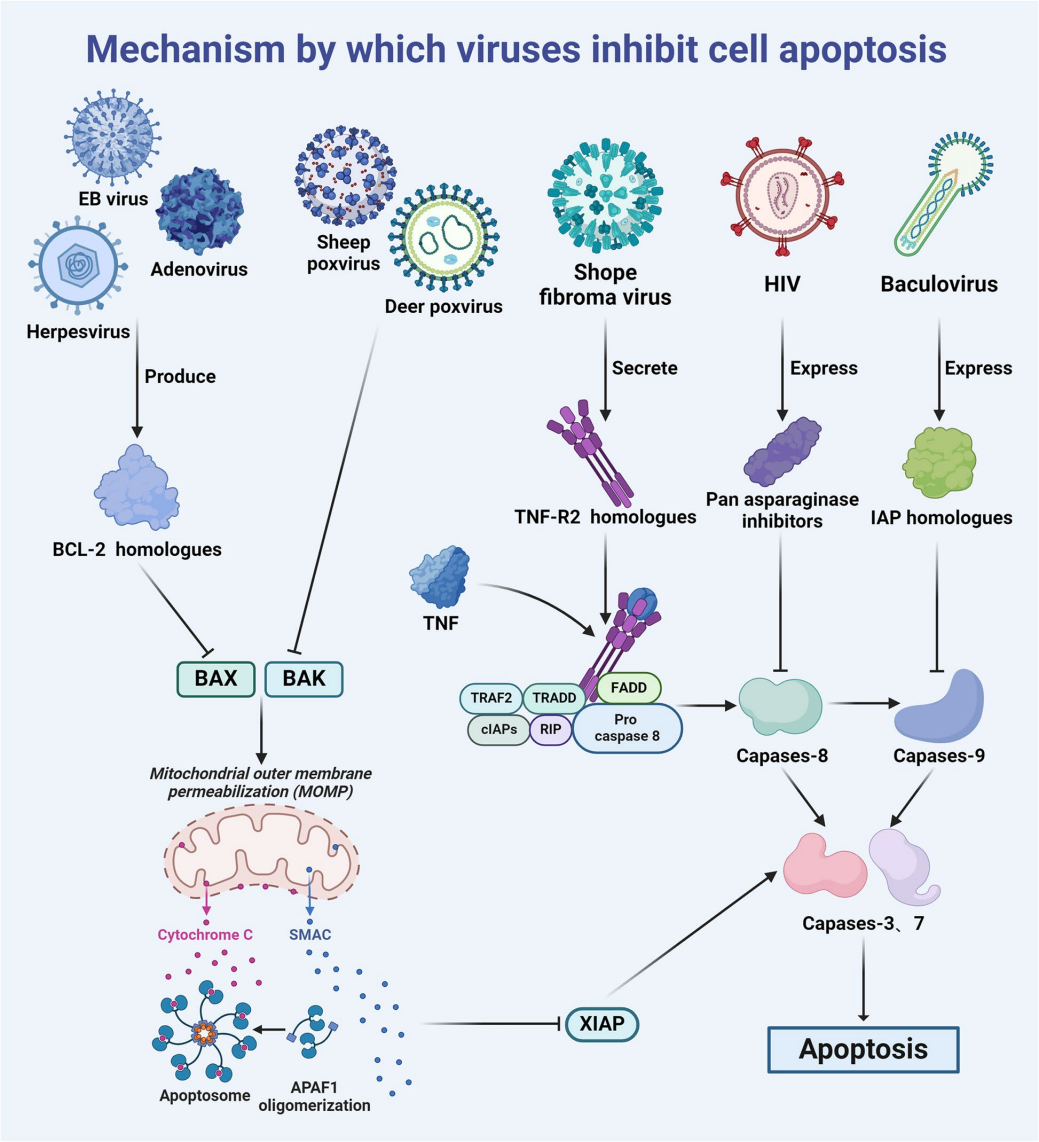
Mechanism by which viruses inhibit cell apoptosis

After invading the host, viruses use various strategies to inhibit apoptosis, thereby facilitating their own survival and reproduction. Different viruses inhibit host cell apoptosis through different pathways. First, they can inhibit the activity of the proapoptotic proteins BAX and BAK. For example, sheep pox [18] and deer pox virus [19] directly inhibit the activity of BAX and BAK to suppress cell apoptosis, while some viruses indirectly inhibit BAX and BAK activity by producing BCL-2 homologues [20]. Examples of such viruses include adenoviruses [21], Kaposi sarcoma-associated herpesvirus [22], and Epstein–Barr virus [23]. A second strategy involves blocking death receptor signalling. One of the earliest documented viral escape mechanisms involves the Shope fibroma virus, which secretes TNF-R2 homologues, neutralizes TNF-α and inhibits cell apoptosis [24]. Finally, another prominent mechanism by which viruses inhibit cell apoptosis is by inhibiting the activity of caspases. For instance, baculoviruses inhibit the activity of caspase-9 by expressing IAP homologues [25], HIV invasion can inhibit the activity of host caspases to prevent Fas-mediated cell apoptosis [26], and the expression of pan-asparaginase inhibitors can inhibit the activity of caspases [27] (Fig. 1).

As shown in this illustration, different viruses use various mechanisms to inhibit apoptosis after entering cells. This inhibition can extend the lifespan of infected cells, thereby promoting viral replication and dissemination within the host.

## The mechanism by which viral invasion activates ZBP1

### Discovery and structure of ZBP1

In 1999, researchers used IFN-γ to stimulate tumour-bearing mice and discovered a new IFN-stimulated gene (ISG). This gene was significantly overexpressed in stimulated mouse tumour stromal cells and macrophages. The researchers named this gene DLM-1 [28]. Subsequent investigations revealed a Z-DNA binding domain in the gene’s N-terminus. Consequently, the research community included this gene in the Z-DNA binding family and named it ZBP1. While early studies did not extensively expand upon the precise functions of ZBP1, there were preliminary speculations that ZBP1 might play a crucial role in both viral invasion and host defence mechanisms [29].

**Fig. 2:**
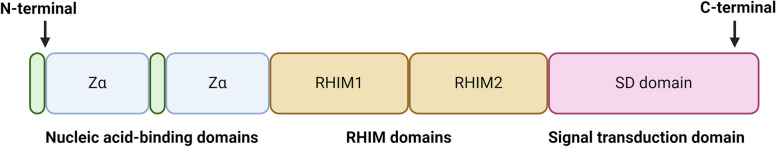
Structure of ZBP1

Subcellular localization studies further revealed that ZBP1 was predominantly found in the cytoplasm, where it could interact with stress granules (SGs) [30]. These findings suggest that ZBP1 may be involved in mRNA metabolism. Subsequently, Taniguchi and colleagues established ZBP1 as a unique DNA sensor capable of recognizing exogenous Z-DNA and mediating innate immune responses [31]. ZBP1 has two N-terminal Zα domains that can bind Z-DNA, two RIP homologous interaction motif domains (RHIM1 and RHIM2) and one C-terminal signal domain (SD) [29, 32]. Further research revealed that the Zα structural domain can bind to not only Z-DNA but also Z-RNA. Moreover, the RHIM domain of ZBP1 facilitates interactions with other RHIM-containing proteins, forming the basis of ZBP1’s signal transduction mechanisms [33, 34]. SD, on the other hand, plays a pivotal role in recruiting and binding to TANK- binding kinase-1 (TBK1), thereby stimulating the production of type I IFN (IFN-I) [35] (Fig. 2).

ZBP1 is characterized by two Zα domains, two RHIM domains and one SD domain. The Zα domain is responsible for binding Z-nucleic acid, while the RHIM domain and SD domain play crucial roles in activating downstream molecules and initiating signal transduction.

### Direct activation of viral Z-nucleic acid

The Zα domain of ZBP1 binds to Z-nucleic acid (Z-NA), thereby initiating downstream signalling pathways that trigger the production of IFN-I and necroptosis. Can nucleic acids activate ZBP1 in response to invasion and initiate an innate immune inflammatory response? Z-RNA [36, 37] and nascent RNA [38, 39] can directly bind to the Zα domain of ZBP1 and activate it. Reports indicate direct sensing of influenza A virus (IAV) RNA by ZBP1 during IAV infection, which activates receptor-interacting protein kinase 3 (RIPK3)-dependent necroptosis. Cells lacking ZBP1 exhibit resistance to IAV-induced cell death [40]. COVID-19 infection results in the cytoplasmic accumulation of Z-RNA, thereby activating the ZBP1-RIPK3 pathway and prompting an inflammatory response that leads to lung injury [41]. In response to Zika virus (ZIKV) infection, the central nervous system activates ZBP1 and downstream RIPK1 and RIPK3 [42]. Unlike other pathways, ZIKV-induced RIPK3 activation does not trigger mixed lineage kinase domain like protein (MLKL)-mediated necroptosis but does inhibit virus proliferation in neural cells by upregulating the expression of the IRG1 enzyme and regulating cell metabolism [42]. Furthermore, the Z-RNA of the vaccinia virus (VACV) can serve as a PAMP, activating the ZBP1-RIPK3 pathway and inducing necroptosis [43]. VACV encodes the E3 protein, which is composed of a N-terminal Z α domain and C-terminal dsRNA binding domain (dsRBD) [44]. Intriguingly, the Zα domain of the E3 protein competitively binds to Z-RNA, inhibiting ZBP1 activation and preventing necroptosis. Conversely, the dsRBD domain stabilizes Z-RNA in the cytoplasm, promoting the accumulation of Z-RNA, which is necessary for ZBP1 activation [43]. Thus, the two distinct functional domains within the E3 protein exert mutual antagonistic effects on ZBP1 activation. A virus lacking the complete E3 region fails to induce ZBP1 activation and subsequent necroptosis. However, mutations resulting in E3 alterations, particularly those affecting the Z α domain, can trigger necroptosis, thereby facilitating viral clearance. This finding shows why E3 Z α-mutated VACV strains are nonpathogenic [45]. Consequently, the viral Z-NA is a direct contributor to the activation of ZBP1.

Only nucleic acids in the Z conformation can activate ZBP1, and viral nucleic acids do not adopt a Z conformation. Therefore, how do these nucleic acids activate ZBP1? At present, the mechanism by which viral nucleic acid activates ZBP1 has not yet been determined. Here, we provide possible explanations. At present, only some viruses, such as IAV [36] and VACA [43], have been shown to bind ZBP1 through Z-RNA. Some viruses, such as mouse cytomegalovirus (MCMV), can only bind to ZBP1 through their endogenous RNA [38]. However, whether these RNA molecules are arranged in the Z conformation is still unclear and requires further experimental testing. Interestingly, GC-rich nucleic acids adopt the Z conformation in test tubes under high-salt conditions, and ZBP1 can bind to GC-rich sequences [46, 47]. Therefore, we hypothesize that in cells, these nucleic acids do not necessarily exist in the Z conformation but are induced into the Z conformation when they bind to ZBP1 and other ZBD-containing proteins. Notably, ZBP1 can recognize both single-stranded RNA and double-stranded RNA. Since MCMV is a DNA virus, when this virus infects cells, it can form double-stranded RNA, which can be recognized by ZBP1 [48]. When endogenous RNA is recognized (such as ribosomal RNA), this single-stranded RNA will fold back when binding to ZBP1 and form a base-pairing hairpin structure that can be recognized by ZBD [49]. In summary, viral nucleic acids can activate ZBP1, but the specific mechanism is not yet clear and requires further investigation.

### Indirect activation of ZBP1 through caspase inhibition

As previously mentioned, viruses can thwart cell apoptosis by inhibiting caspase activity. However, intracellular caspases inhibit innate immune sensors. These factors can cleave immune sensors, maintaining them in a state of low activity to prevent the occurrence of autoimmune reactions [50]. When caspase activity is inhibited, immune sensors, including ZBP1, can prevent caspase cleavage, thereby activating the innate immune response. Studies have revealed a significant increase in ZBP1 expression when caspase-8 is either absent or when its activity is decreased in cells. This subsequently triggers the activation of RIPK3 and MLKL, culminating in necroptosis [51]. After treatment with interferon, caspase-8-deficient cells are more susceptible to ZBP1-induced necroptosis than normal cells [52]. Even though the virus inhibits apoptosis by producing a caspase inhibitor, necroptosis induced by ZBP1 is a competent alternative to cell apoptosis for pathogen clearance. Although the virus wants to evade clearance from the body by inhibiting apoptosis, ZBP1, which is subsequently activated by caspase-8 inhibition, induces a robust innate immune response, effectively eliminating invading pathogens through an alternative pathway.

### Destruction of mitochondrial membrane integrity resulting in mtDNA release

The invasion of numerous viruses has been linked to impaired mitochondrial function. Current research has shown that the envelope protein gp120 of human immunodeficiency virus 1 (HIV) can compromise the integrity of inner mitochondrial membrane cristae [53]. Additionally, the viral protein R (Vpr) produced by HIV has been shown to concentrate the mitochondrial matrix, leading to mitochondrial constriction, the induction of swollen cristae, and changes in the outer mitochondrial membrane (OMM) with concomitant mitochondrial endoplasmic reticulum (ER) contact degradation [54]. Furthermore, the p13 protein of human T-cell leukaemia virus 1 (HTLV-1) can induce mitochondrial swelling and lysis [55]. The PB1-F2 protein of the influenza virus is known to trigger disruptions in mitochondria [56]. Nucleic acids within mitochondria, such as mitochondrial DNA (mtDNA) and other dsRNAs, serve as damage-associated molecular patterns (DAMPs) that can initiate innate immune and inflammatory responses [57, 58]. Under normal physiological conditions, these nucleic acids reside within mitochondria and remain secluded from innate immune sensors within the cytoplasm. However, under specific pathological conditions, such as the invasion of pathogens, these nucleic acids can enter the cytoplasm when the mitochondrial membrane is compromised [59], where they activate ZBP1 and other innate immune sensors, thereby initiating downstream inflammatory pathways [60].

However, ZBP1 can be activated only by Z-DNA, and mtDNA is similar to traditional DNA in that it is in a B-conformation rather than a Z-conformation. In this case, how does mtDNA released into the cytoplasm activate ZBP1? Studies have shown that viral invasion can induce mtDNA stress, causing instability of the mtDNA structure [61]. The instability of mtDNA induces the accumulation of Z-DNA [62]. After these Z-DNAs are released into the cytoplasm, they can be stabilized by ZBP1 and subsequently activate ZBP1, which cooperates with cGAS to initiate IFN-I signalling. Therefore, it is not mtDNA itself that directly activates ZBP1; instead, mtDNA stress caused by viral invasion and subsequent Z-DNA accumulation indirectly activates ZBP1 and initiates the innate immune response. In addition, as mentioned above, ZBP1 and other ZBD proteins can induce nucleic acids to adopt the Z conformation when they bind to them. Therefore, we hypothesize that mtDNA may also be induced to adopt the Z conformation when binding to ZBP1, thereby activating ZBP1.

As highlighted earlier, cellular stress can induce apoptosis through the mitochondrial pathway, potentially causing the release of mtDNA from the mitochondrial outer membrane [63]. However, under normal conditions, cells are typically immunologically quiescent and devoid of inflammatory reactions. In contrast, when pathogenic invasion leads to mtDNA release, a robust inflammatory response ensues, facilitating viral clearance. What accounts for these diametrically opposed effects? The key disparity lies in the involvement of caspases. Typically, caspases maintain intracellular immune sensors such as ZBP1 in a subdued state, preventing endogenous DNA, such as mtDNA, from inducing IFN-I production [64]. Consequently, the release of mtDNA via intrinsic apoptotic pathways fails to activate ZBP1 or other innate immune nucleic acid sensors, thereby silencing immune responses associated with cell apoptosis. However, pathogen invasion can inhibit caspase activity [7, 50]. Normally, intracellular caspases keep these DNA sensors inert. When a virus infiltrates and inhibits caspase activity, these sensors become active. In response to endogenous DNA, downstream molecules become activated, stimulating IFN-I production. This triggers an inflammatory response, fortifying the innate immune reaction against infection [65].

### Inhibition of ADAR1 indirectly activates ZBP1

**Fig. 3:**
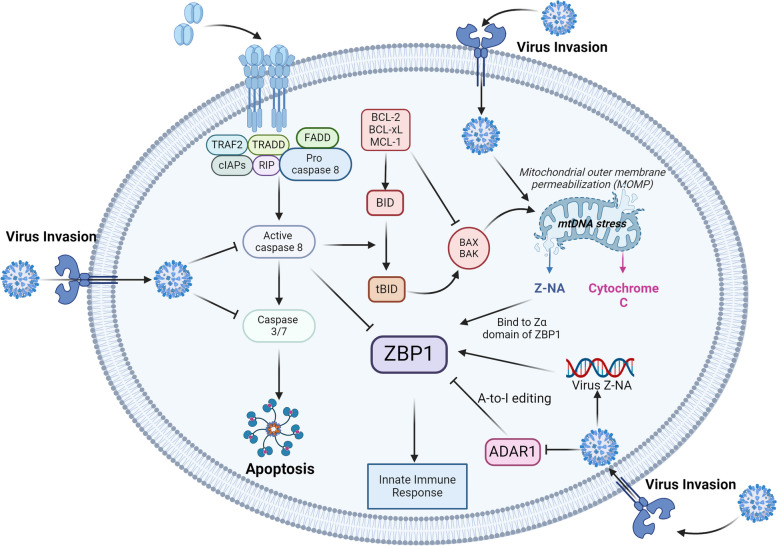
The mechanism by which viral invasion activates ZBP1

Initially recognized as a crucial factor in averting autoimmune reactions, adenosine deaminase 1 (ADAR1) plays a pivotal role in destabilizing double-stranded RNA, consequently decreasing its immunostimulatory properties. Under standard conditions, ADAR1 modifies endogenous RNA, preventing the activation of innate immune nucleic acid sensors [66, 67]. Similar to ZBP1, ADAR1 possesses a Zα structural domain. This domain facilitates A-to-I editing of endogenous Alu elements, which is an abundant RNA modification in the mammalian transcriptome. By inhibiting the formation of double-stranded RNA through reverse pairing of Alu repeat sequences, ADAR1 inhibits the activation of ZBP1, thereby avoiding the onset of autoimmunity [68]. Furthermore, ADAR1 directly inhibits the activation of RIPK3 by competitively binding to ZBP1, thereby inhibiting the occurrence of necroptosis [69]. Mutations in ADAR1 can trigger a cascade of pathological immune reactions by activating ZBP1, establishing a link between ZBP1 activation and pathological responses resulting from ADAR1 mutations [70]. In addition, ADAR1 inhibits the production of IFN-I induced by ZBP1 [71] This inhibitory effect of ADAR1 on ZBP1 establishes a delicate equilibrium in endogenous nucleic acid sensing, but it also hampers the body’s ability to respond to pathogenic invasions, disrupting this balance. Studies have demonstrated that VAI RNA of adenoviruses can suppress the RNA editing activity of ADAR1 [72]. Moreover, the E3L protein produced by the cowpox virus can inhibit the activity of ADAR1 [73]. When ADAR1 activity is inhibited by viral invasion, ZBP1 is consequently activated. This intricate interplay between ADAR1 and ZBP1 highlights the nuanced regulatory mechanisms involved in the host response to autoimmune challenges and pathogenic threats (Fig. 3).

Z-RNA and nascent RNAs of the virus can directly bind to the Zα domain of ZBP1 and activate it. In addition, viruses can damage mitochondrial membranes, causing mtDNA stress. The instability of the mitochondrial genome promotes the accumulation of Z-DNA. When the mitochondrial membrane is damaged by viruses, many types of nucleic acids, such as mtDNA, Z-DNA, and dsRNA, are released into the cytoplasm. When Z-DNA accumulates due to mtDNA stress, it can activate ZBP1. Under normal physiological conditions, ZBP1 inhibitory molecules are present in cells and prevent endogenous nucleic acids from activating ZBP1 and triggering autoimmune reactions. Viral invasion can downregulate ZBP1 inhibitory molecules, such as caspases and ADAR1, thereby indirectly activating ZBP1 and triggering downstream signalling.

## Activation of ZBP1 and initiation of the innate immune response

As mentioned earlier, the activation of ZBP1 occurs when a virus infiltrates cells, and conventional apoptosis mechanisms are inadequate. We will how examine the pathway through which activated ZBP1 orchestrates innate immune responses to mount effective antiviral defences.

### Induction of IFN-I production by ZBP1

**Fig. 4:**
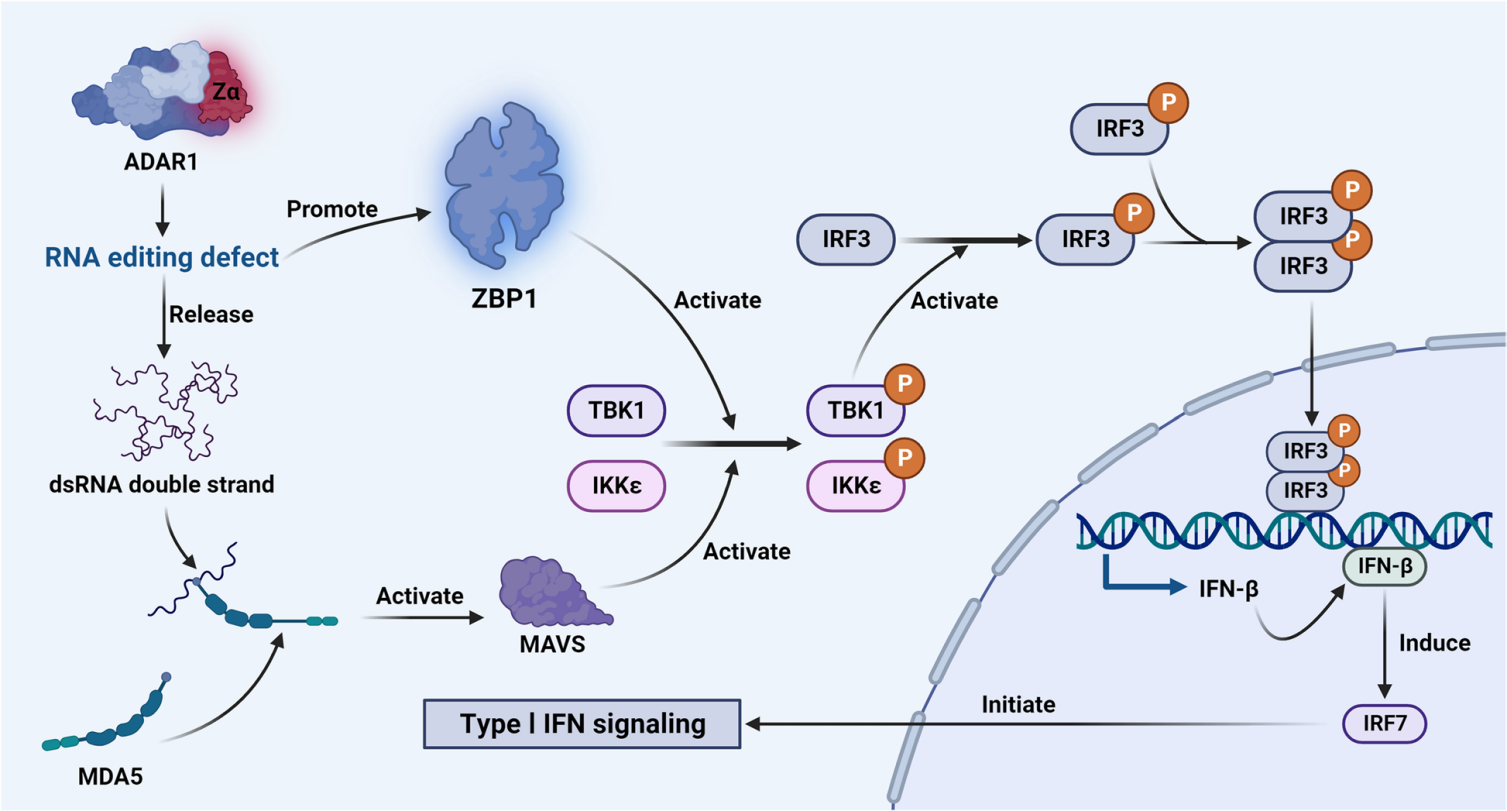
ZBP1-mediated induction of the IFN-I pathway

Initially, ZBP1 was thought to play a crucial role in innate immune responses by triggering the production of IFN-I [28]. ZBP1 overexpression has been shown to significantly increase the expression of IFN-I in mouse embryonic fibroblasts [31]. The mechanism involves the recruitment and phosphorylation of TBK1 by the activated ZBP1 complex, which subsequently recruits IFN regulatory factor 3 (IRF3) for phosphorylation [31, 74] After recruitment, the phosphorylation of adapter proteins by TBK1 and inhibitor of nuclear factor kappa-B kinase epsilon (IKKε) activate the docking site of IRF3 [75]. Subsequently, TBK1- or IKKε-mediated phosphorylation leads to the dissociation of IRF3 from adapter proteins, initiating homodimerization (p-IRF3). This process precedes the transport of p-IRF3 to the nucleus, facilitating IFN-β transcription [76, 77]. The induction of IFN-β further activates IRF7, initiating the signalling cascade of IFN-I [78]. Recent studies propose an alternative pathway for ZBP1-induced IFN-I that primarily involves the absence and disruption of ADAR1. ADAR1, which is an RNA editing enzyme, uses dsRNA as a substrate to convert adenosine into inosine, disrupting GC pairing. When Z-RNA is modified by ADAR1, it is unable to bind and activate ZBP1 [71]. Destruction of the Zα domain of ADAR1 results in RNA editing defects [79]; consequently, the dsRNA is released. These unmodified Z-RNAs can activate ZBP1, initiating IFN-I signalling. In addition, these unmodified Z-RNAs can also be sensed by melanoma differentiation-related protein 5 (MDA5) [80]. MDA5 and retinoic acid-inducible gene I (RIG-I), which are both cytosolic PRRs, activate the adapter protein mitochondrial antiviral phage protein (MAVS) in response to binding to ligands [81]. MAVS, in turn, recruits and activates TBK1 kinase and IKKε. Following recruitment, TBK1 and IKK ε phosphorylate adapter proteins, activating the docking site of IRF3 [75]. This activation event triggers interferon transcription signalling (Fig. 4).

Disruption of the Zα domain of ADAR1 results in defective RNA editing, leading to the release of dsRNA. MDA5 can then bind these dsRNA molecules, initiating downstream activation of MAVS. Activated MAVS subsequently recruits and activates TBK1 and IKKε. Moreover, the RNA editing defects in ADAR1 promote the activation of ZBP1. Following these events, TBK1 or IKKε phosphorylates IRF3 to form a homodimer (p-IRF3), which translocates into the nucleus, promoting the transcription of IFN-β. Ultimately, the induction of IFN-β leads to the production of IRF7, initiating the IFN-I signalling cascade.

### Pyroptosis

**Fig. 5:**
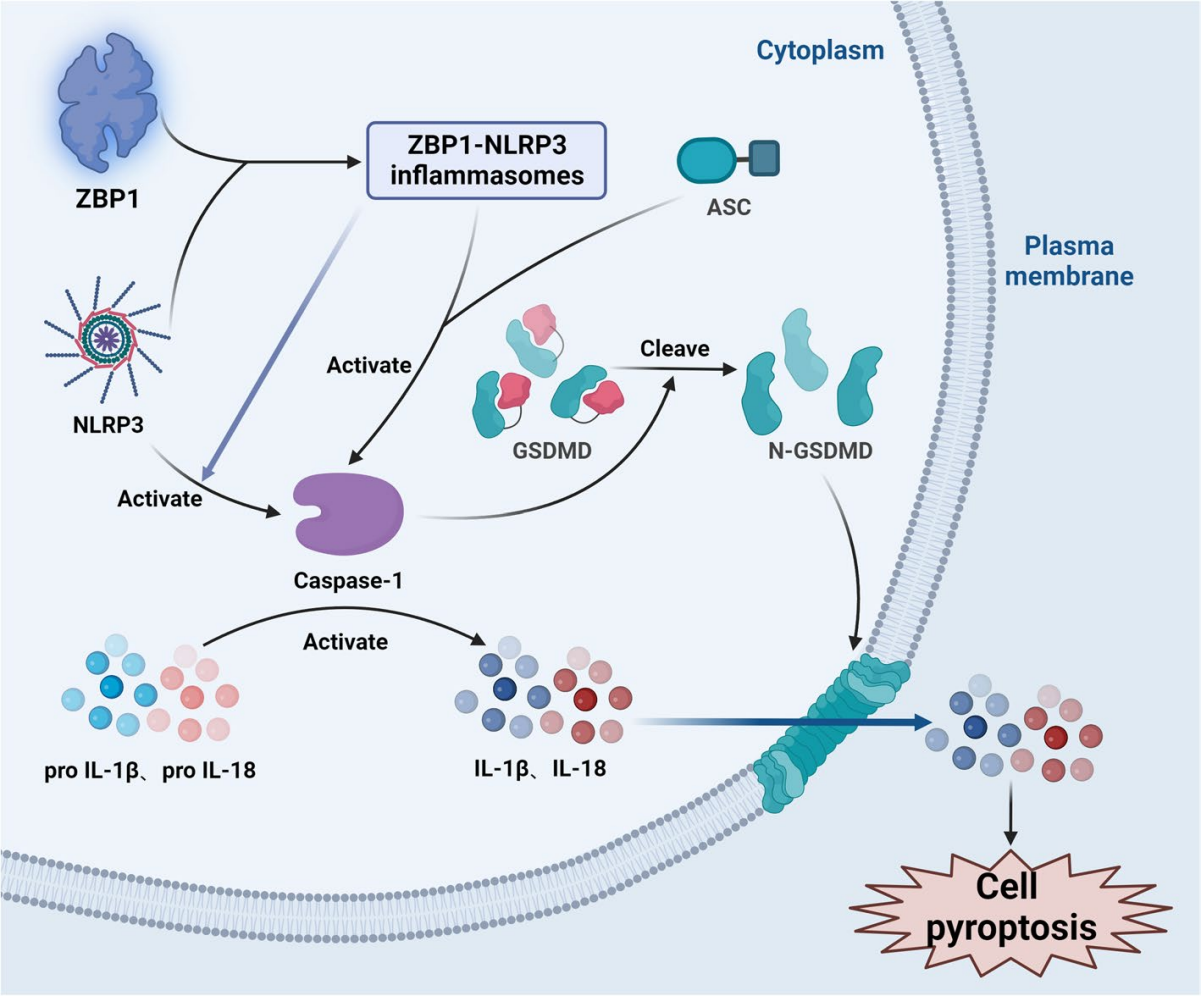
Mechanism of ZBP1-induced pyroptosis

ZBP1 plays a pivotal role in stimulating cell lysis and inflammation through pyroptosis. Notably, apoptosis, which is a programmed cell death process, is activated by inflammasomes in response to injury and infection [82]. Pyroptosis is characterized by the ability of the gasdermin family to induce cell membrane pore formation, cell membrane destruction, and the release of proinflammatory cytokines. Taking gasdermin D (GSDMD) as an example, during IAV infection, the activation of nucleoside binding domain and leucine-rich repeat family pyrin domain containing 3 (NLRP3) inflammasomes is completely dependent on ZBP1. ZBP1 directly activates NLRP3, leading to the formation of ZBP1-NLRP3 inflammasomes. In response to activation of the ZBP1-NLRP3 inflammasome [83], NLRP3 triggers the activation of caspase-1, which in turn hydrolyses the cytokine IL-1 through protein hydrolysis. Furthermore, the precursor form of IL-18 is activated [84]. Caspase-1 cleaves GSDMD into N-terminal fragments during this process, resulting in the formation of pores in the plasma membrane. Subsequently, the activated proinflammatory cytokines IL-1β and IL-18 in the cytoplasm are released to the outside of the cell through pores in the cell membrane [85, 86]. In addition, the released cell contents act as PAMPs and DAMPs in surrounding cells, further intensifying the inflammatory response [82]. In addition to its role in activating pyroptosis, the ZBP1-NLRP3 inflammasomes can directly initiate inflammatory responses. ZBP1-NLRP3 binds to apoptosis-related receptors containing CARD (ASC) adapter molecules, directly activating caspase-1, which in turn activates and releases IL-1β and IL-18, thereby inducing an inflammatory response [83] (Fig. 5).

The ZBP1-NLRP3 inflammasome, which is formed by the binding of ZBP1 to NLRP3, can activate caspase-1 directly or by interacting with ASC. Once activated, caspase-1 initiates the cleavage of the precursors of IL-1β and IL-18. Furthermore, caspase-1 cleaves GSDMD into its N-terminal fragment (N-GSDMD). This N-GSDMD fragment punctures the cell membrane, triggering the release of IL-1β and IL-18 from the cell and ultimately inducing pyroptosis.

### Necroptosis

**Fig. 6:**
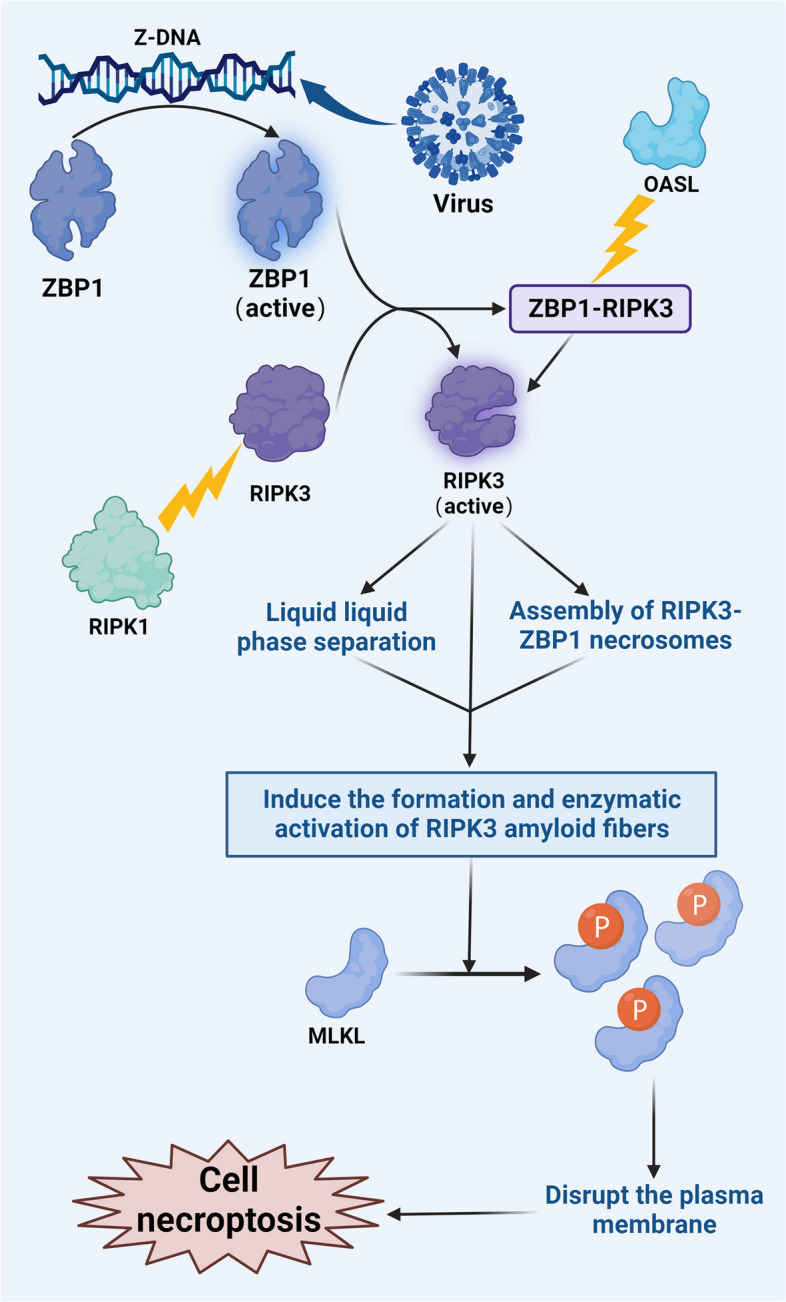
Mechanism of ZBP1-induced necroptosis

Necroptosis has been regarded as an unintended outcome of severe external damage that can cause cellular harm. ZBP1 can stimulate inflammatory reactions through necroptosis. ZBP1 possesses two Zα domains, Zα 1 and Zα 2, which function as binding sites for Z-DNA and become activated in response to binding with Z-DNA. The interaction between ZBP1 and RIPK3 occurs through the RHIM-RHIM interaction and involves the RHIM domain of ZBP1 and the RHIM domain of RIPK3. This interaction results in the formation of a complex that riggers immediate oligomerization and self-phosphorylation of RIPK3 [82]. Liquid‒liquid-phase separation (LLPS) is a novel model of the formation of biofilm-free biomolecular aggregates, enabling the regulation of biochemical signals. Interferon-induced 2'-5' oligoadenylate synthetase-like protein (OASL) acts on the ZBP1-RIPK3 complex through its N-OAS and C-UBL domains, thereby activating RIPK3. This activation leads to LLPS and facilitates the assembly of RIPK3-ZBP1 necrosomes [87, 88]. These phase condensates serve as platforms for RIPK3 nucleation, culminating in the formation and enzymatic activation of RIPK3 amyloid fibres. Subsequently, there is high phosphorylation and oligomerization of MLKL among BCL-2 family proteins, resulting in the formation of ion gradients, water influx, cell swelling, and osmotic dissolution, ultimately culminating in necrosis [84, 89]. In addition to ZBP1, receptor-interacting protein kinase 1 (RIPK1) can phosphorylate downstream RIPK3 through its RHIM domain, thereby activating MLKL and inducing necroptosis [90]. However, RIPK3 activation is kinase dependent. In the absence of a kinase, RIPK1 can bind to RIPK3 through its RHIM domain but cannot promote its phosphorylation or activation. Consequently, ZBP1 is unable to bind to RIPK3, competitively inhibiting the activation of RIPK3 by ZBP1 and preventing RIPK3 activation and necroptosis [91]. RIPK1 can competitively bind to ZBP1, inhibiting RIPK3-mediated necrosis [43, 75]. How does ZBP1 trigger inflammation after inducing necroptosis? In the nervous system, necroptosis in microglia is a hallmark of nervous system inflammation. Following necroptosis, microglia can induce the secretion of key proinflammatory cytokines, such as TNF-α, IL-1β, and MCP-1, thereby promoting neuroinflammation in the diabetic retina [92]. Moreover, the ZBP1/RIPK3/caspase-8 complex can stimulate the formation of the NLRP3 inflammasome. In turn, NLRP3 can sense dsDNA or mtDNA, inducing the formation of inflammatory red blood cells, Gasdermin D cleavage and the creation of Gasdermin D pores leads to the release of mature IL-1β and subsequent inflammation [93]. During IAV infection, MLKL can promote the activation of the NLRP3 inflammasome and the release of IL-1 through potassium efflux, thereby contributing to inflammation [94]. In the context of pyroptosis, we discussed inflammation caused by the activation of the NLRP3 inflammasome and the initiation of pyroptosis. In other words, necrosis can further trigger pyroptosis by activating NLRP3, illustrating crosstalk between pyroptosis and necroptosis (Fig. 6).

Activated ZBP1 forms a complex with RIPK3 termed the ZBP1-RIPK3 complex. OASL, in turn, acts on ZBP1-RIPK3 to facilitate the assembly of RIPK3–ZBP1 necrosomes, leading to the formation and enzymatic activation of RIPK3 amyloid fibrils. Subsequently, MLKL undergoes substantial phosphorylation, resulting in the creation of ion channels that compromise the integrity of the plasma membrane and trigger necroptosis. Furthermore, although RIPK1 cannot activate RIPK3, it can bind to RIPK3. Instead, it competitively inhibits the binding of RIPK3 to inhibit ZBP1-mediated necroptosis.

### ZBP1 restarts the apoptotic signal

Apoptosis is the primary mechanism through which cells combat pathogen infection. Apoptosis is mainly mediated by caspases and leads to cell rupture and death, and apoptotic cells are subsequently engulfed to prevent pathogen spread [95]. As previously mentioned, viral interference inhibits cell apoptosis by suppressing caspase activity. However, the interplay between necrosis and apoptosis can reignite the apoptosis process. Within the necrosis mechanism, OASL facilitates the coalescence of RIPK3 and ZBP1 within droplets through RHIM-mediated interactions. This leads to rapid homologous oligomerization of RIPK3, which results in the formation of powdery fibrils. The resultant high-order assembly, which is an amyloid fibre, functions as a platform to induce signal transduction. It increases the expression of specific apoptotic-like proteins, such as ASC, FAS-associated death domain (FADD), and caspase-8, thereby rekindling apoptosis [89]. The inhibition of caspase-8 is ameliorated, reigniting apoptosis. This finding shows that RIPK3 is not solely responsible for inducing necrosis and subsequent inflammation but also facilitates ZBP1-RIPK1-mediated apoptosis. However, certain studies have shown that even in the absence of RIPK3, ZBP1 can still initiate caspase-8-dependent apoptosis [70]. This finding suggests that while RIPK3 can promote cell apoptosis, its presence is not a strict prerequisite for activating apoptosis. Therefore, how does the reactivation of apoptosis contribute to an inflammatory response? ZBP1 plays a pivotal role in this process by recruiting both RIPK3 and RIPK1, which form a proinflammatory complex containing ubiquitin ligases and deubiquitinases of the K63‐ and M1‐linked polyubiquitin machinery. These proinflammatory complexes mediate inflammatory signal transduction. Specifically, the ubiquitination of RIPK1, which is orchestrated by ZBP1, promotes kinetic, mitogen-activated protein kinase (MAPK) and nuclear factor-κB (NF-κB) signal transduction, leading to the activation of IFN-I and other proinflammatory factors [86, 96]. Interestingly, caspase-8 that is generated during apoptosis has been shown to inhibit inflammatory signalling via the ZBP1-RIPK3 pathway, contributing to the relatively mild level of inflammation induced by apoptosis [97]. Furthermore, caspase-8 can activate caspase-3, which cleaves cGAS [50] This process inhibits the ZBP1-cGAS-STING pathway, further inhibiting the inflammatory response and adding another layer to the moderation of inflammation resulting from apoptosis. Additionally, the pores created by GSDMD-N fragments on the cytoplasmic membrane lead to the efflux of potassium ions, activation of the NLRP3 inflammasome and the release of IL-1β, all of which contribute to inflammation [94]. There is a significant degree of crosstalk between pyroptosis and apoptosis. The upstream regulation of NLRP3 is mediated by the RIPK1-RIPK3-caspase-8 axis, which is governed by ZBP1 [86]. This finding suggests that the upregulation of caspase-8, which is a driver of apoptosis, can promote pyroptosis. During apoptosis, caspase-8 can directly cleave GSDMD into N-terminal fragments, while during pyroptosis, the pores formed by GSDMD-N fragments in the cytoplasmic membrane lead to potassium ion efflux. Furthermore, this process triggers the activation of the NLRP3 inflammasome, stimulating pyroptosis and further amplifying the inflammatory response. This intricate interplay underscores the occurrence of regulatory crosstalk between apoptotic and pyroptotic cells during the overall apoptotic process. These insights into crosstalk offer promising implications for modulating the inflammatory response mediated by ZBP1.

### Crosstalk between the ZBP1-induced inflammatory pathway and the cGAS-STING pathway

**Fig. 7:**
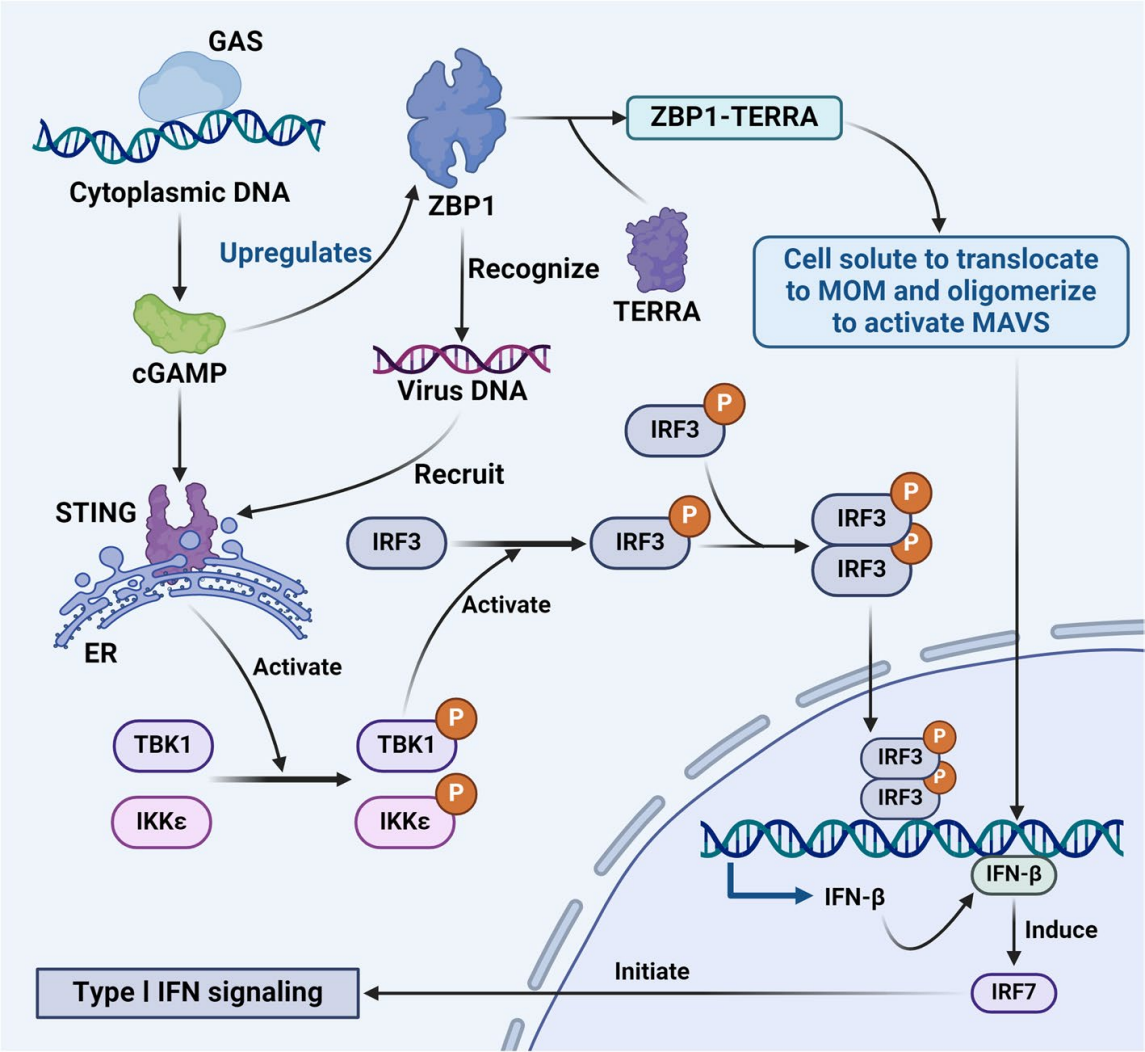
Crosstalk between the ZBP1-induced inflammatory pathway and the CGAS-STING pathway

The cGAS-STING signalling pathway is an innate immune mechanism in which cGAS acts as a GMP-AMP synthase. As a sensor for cytoplasmic DNA, cGAS catalyses the synthesis of the second messenger cGAMP. Moreover, stimulator of interferon genes (STING) facilitates the autophosphorylation of TBK1 by binding to cGAMP, leading to oligomerization. This complex then translocates from the endoplasmic reticulum to the Golgi apparatus. STING, which is phosphorylated by TBK1, recruits IRF3, initiating dimerization and translocation to the nucleus and ultimately mediating the transcription of IFN-I [98]. Recent investigations have shown the capacity of ZBP1 to induce INF production and inflammation through the cGAS-STING pathway. In the cGAS-STING sensor pathway, DNA sensing by cGAS-STING directly upregulates the expression of ZBP1. ZBP1 is fully activated in response to binding to the transcriptional product of telomeric repeat RNA (TERRA), which is synthesized by malfunctioning telomeres. This binding induces ZBP1 to undergo oligomerization, during which the protein is transformed into filamentous substances on the outer membranes of mitochondria. Subsequently, conformational changes occur, prompting the translocation of solutes to the mitochondrial outer membrane (MOM), where they oligomerize to activate MAVS. This activation further induces the transcription of IFN-β, triggering IFN-I signalling and culminating in inflammation [99]. In the aforementioned pathways, ZBP1 plays a pivotal role in IFN-I activation by phosphorylating adapter proteins such as MAVS. This finding prompted us to examine whether ZBP1 could activate IFN-I signalling through the STING pathway as a potential bridging protein. Interestingly, receptors such as IFN-γ-inducing protein 16 (IFI16) and ZBP1, which are involved in recognizing double-stranded viral DNA, can recruit STING as adapter molecules. This, in turn, activates the TBK1 and IRF3 pathways, mediating the production of IFN-I and consequent inflammation [100]. Previously, we noted that ZBP1 induced IFN-1 signalling. Notably, IFN-1 reciprocated by promoting the expression of ZBP1. The ISG pathway, which is activated downstream of IFN-1, initiates immune defence mechanisms. ZBP1 is an ISG that creates a natural feedback loop in which IFN-1 increases the expression of ZBP1 [52]. In the context of human cytomegalovirus (HCMV) infection, ZBP1 amplifies HCMV-induced IRF3 activation and IFN-β expression. Moreover, IFN-β-induced JAK/STAT signalling strongly upregulates ZBP1 [101, 102]. This interaction establishes a positive feedback loop between ZBP1 and IFN, augmenting the innate immune response and maintaining the body’s overall immune defence. Moreover, in the context of radiotherapy and radiation, RHIM in the N-terminus of ZBP1 binds with RIPK3 to form RIPK3–ZBP1 necrosomes. Subsequently, RIPK3 phosphorylates mixed lymphocyte kinase-like proteins, facilitating the fusion of nuclear and cytoplasmic membrane pores and ultimately leading to necrosis. Furthermore, the ZBP1-MLKL necrosis cascade induces the accumulation of cytoplasmic mtDNA in irradiated tumour cells. This accumulation of mtDNA activates the cGAS-STING signalling pathway, thereby governing the expression of IFN-I in response to radiation [60]. This process triggers an inflammatory response. This interaction represents crosstalk between necrosis and the IFN-induced inflammatory pathway, illustrating their interconnectedness and mutual influence. The cGAS-STING pathway serves as link between necrosis and IFN production that stimulates inflammation, which is clinically relevant (Fig. 7).

The crosstalk between the ZBP1-induced inflammatory pathway and the cGAS-STING pathway is dynamic. In the cGAS-STING pathway, cytoplasmic DNA is sensed by cGAS, leading to the synthesis of cGAMP. Subsequently, cGAMP associates with STING, inducing its oligomerization. This event prompts TBK1 autophosphorylation. Phosphorylated STING is recognized and phosphorylated by TBK1, which results in the formation of the STING-TBK1 complex. This complex then recruits and phosphorylates IRF3, which translocates into the nucleus, promoting the transcription of IFN-β. The resulting IFN-β induces the production of IRF7, initiating the type I IFN signalling cascade. In the DNA-sensing pathway cGAS–STING, ZBP1 expression is upregulated. ZBP1, in turn, binds to the TERRA transcript, facilitating oligomerization in the cytosol the translocation and to the MOM. This activation of MAVS promotes the transcription of IFN-β, leading to the production of IRF7 and the initiation of IFN-I signalling. Additionally, double-stranded viral DNA is recognized by IFI16 and ZBP1/DAI receptors, which recruit STING. Activated STING then engages the TBK1 and IRF3 pathways, facilitating the transcription of IFN-β. Ultimately, IFN-β induces the production of IRF7, initiating the type I IFN signalling cascade. This intricate crosstalk highlights the interconnected and synergistic nature of these signalling pathways with the host antiviral response.

### Positive feedback regulation in the ZBP1-induced inflammation pathway

Positive feedback regulation is critical for the innate immune response and plays a crucial role in amplifying the innate immune response. This circuit not only directly magnifies the inflammatory reaction but also fortifies the overall immune response, enabling the body to effectively combat infection [103]. Leveraging these positive feedback pathways as regulatory targets is clinically valuable for modulating the innate immune response. This control aids in managing the extent of both inflammatory and antiviral responses. For example, in T cells lacking replication protein A1 (RPA1), the leakage of genomic DNA into the cytoplasm activates the ZBP1-RIPK3 pathway, ultimately leading to necrosis and apoptosis. After necroptosis, DAMPs are released, and they recruit white blood cells and further exacerbate the inflammatory response [104]. Similarly, during IAV infection, macrophages induce further proinflammatory activity and necroptosis. Necroptosis, in turn, results in the production of nitric oxide, IL-6, and TNF, giving rise to inflammatory storms. This cascade effect further amplifies inflammation, establishing a positive feedback loop that regulates the inflammatory pathway [105]. Strategically managing these feedback mechanisms is an avenue for controlling and fine-tuning the innate immune response and provides a valuable approach for clinical intervention. In addition, we mentioned earlier that ZBP1 not only induces IFN-1 signalling but also reciprocally influences IFN-1 signalling. The ISG pathway, which is activated downstream of IFN-1, is a catalyst for immune defence. ZBP1, which is an ISG, is naturally promoted by IFN-1, establishing a bidirectional relationship [52]. In the context of HCMV infection, ZBP1 enhances HCMV-induced IRF3 activation and IFN-β expression. Concurrently, IFN-β-induced JAK/STAT signalling robustly upregulates ZBP1 [101, 102]. This reciprocal interaction between ZBP1 and IFN results in the formation of a positive feedback circuit that actively promotes the innate immune response. This circuit holds immense importance for the overall immune response.

## Conclusion

Apoptosis serves as the primary mechanism by which the body eliminates viruses. Infected cells can transmit signals that initiate apoptosis, resulting in the subsequent clearance of pathogens and thereby preserving the internal environment. However, viruses have evolved mechanisms to inhibit cell apoptosis, evade elimination and persist within host cells. When apoptosis is suppressed, the nucleic acid sensor ZBP1 becomes pivotal, activating innate immune responses to clear pathogens. ZBP1 directly detects viral nucleic acids and initiates downstream pathways. This review comprehensively outlined the signalling pathways and crosstalk involving ZBP1, including ZBP1-induced IFN-I, pyroptosis, and necroptosis. Downstream molecules activated by ZBP1 play crucial roles in reactivating apoptotic signals that are suppressed by viruses through diverse mechanisms, thus reinstating apoptosis. Furthermore, our investigation revealed that the pathway activated by ZBP1 interferes with the cGAS-STING pathway. The inflammatory signalling cascade mediated by ZBP1 exhibits positive feedback regulation, aiding in the activation of inflammatory pathways for pathogen clearance. However, it is imperative to note that this regulatory effect is a double-edged sword. Excessive activation of positive feedback can trigger a cytokine storm, resulting in tissue and organ damage [106]. Balancing the activation of these pathways is crucial for harnessing the benefits of the immune response while mitigating potential harm.

Optimal ZBP1 activity is crucial, as both excessive and insufficient ZBP1 levels yield undesirable outcomes. Research indicates that reducing ZBP1 expression in the lungs of mice can attenuate pulmonary inflammatory responses during infection. Paradoxically, this leads to a significant increase in viral load in the lungs, exacerbating the infection. Despite an increased viral presence, the overall prognosis of mice is improved [107]. Similar trends have been observed in various animal models, highlighting that tissue and organ damage arising from an excessive inflammatory response rather than from the viral infection itself constitutes the primary cause of death [108–111]. However, contradictory findings exist. Thapa et al. reported that decreased ZBP1 in mice weakened the inflammatory response and resulted in substantial viral accumulation and increased mortality [40]. This discrepancy might stem from variations in the experimental models used among studies. Clinically, the interplay between inflammatory responses and viral infections varies among individuals. The key challenge lies in orchestrating the innate immune inflammatory response to eradicate pathogens without causing undue harm to tissues or organs. The prospect of designing drugs targeting the ZBP1 signalling pathway holds promise. These drugs could serve as dual-purpose solutions for effectively combating infections while mitigating inflammatory damage, which would be a potential breakthrough in public health.

## Data Availability

No datasets were generated or analysed during the current study.

## Abbreviations

ZBP1: Z-DNA binding protein 1
BCL-2: B-cell lymphoma-2
PAMPs: Pathogen associated molecular patterns
PRRs: Pathogen recognition receptors
IFN: Interferon
BAX: Bcl-2 associated X Protein
BAK: Bcl-2 Antagonist/Killer
MOMP: Mitochondrial outer membrane and permeabilize
ISG: Interferon-stimulated gene
SGs: Stress granules
SD: Signal domain
TBK1: TANK-binding kinase 1
IFN- I: Type I IFN
Z-NA: Z-nucleic acid
IAV: Influenza A viruses
RIPK3: Receptor-interacting protein kinase 3
ZIKV: Zika virus
MLKL: Mixed lineage kinase domain like protein
VACV: Vaccinia virus
dsRBD: DsRNA binding domain
HIV: Human immunodeficiency virus 1
Vpr: Viral protein R
OMM: Outer mitochondrial membrane
ER: Endoplasmic reticulum
HTLV-1: Human T-cell leukaemia virus 1
mtDNA: Mitochondrial DNA
DAMP: Damage related molecular pattern
IRF3: IFN regulatory factor 3
IKKε: Inhibitor of nuclear factor kappa-B kinase epsilon
MDA5: Melanoma differentiation related protein 5
MAVS: Mitochondrial antiviral phage protein
RIG-I: Retinoic acid-inducible gene
p-IRF3: Phosphorylates IRF3
GSDMD: Gasdermin D
NLRP3: Nucleoside binding domain and leucine rich repeat family pyrin domain containing 3
ASC: Apoptosis related receptors containing CARD
N-GSDMD: GSDMD into its N-terminal fragment
LLPS: Liquid–liquid phase separation
OASL: Oligoadenylate synthetase like protein
RIPK1: Receiver interacting protein kinase 1
FADD: FAS-associated death domain
MAPK: Kinetics and mitogen activated protein kinase
NF: κ B: Nuclear factor- κ Appa B
TERRA: Telomeric repeat RNA
MOM: Mitochondrial outer membrane
IFI16: Interferon- γ Inducing protein 16
HCMV: Human cytomegalovirus
